# Post-Secretion Processes and Modification of Extracellular Vesicles

**DOI:** 10.3390/cells14060408

**Published:** 2025-03-11

**Authors:** Artem Ten, Natalia Yudintceva, Konstantin Samochernykh, Stephanie E. Combs, Hem Chandra Jha, Huile Gao, Maxim Shevtsov

**Affiliations:** 1 Laboratory of Biomedical Nanotechnologies, Institute of Cytology of the Russian Academy of Sciences (RAS), 194064 Saint Petersburg, Russia; ten.arteym@gmail.com (A.T.); yudintceva@mail.ru (N.Y.); 2 Personalized Medicine Centre, Almazov National Medical Research Centre, 2 Akkuratova Str., 197341 Saint Petersburg, Russia; samochernykh_ka@almazovcentre.ru; 3 Department of Radiation Oncology, Technishe Universität München (TUM), Klinikum Rechts der Isar, Ismaninger Str. 22, 81675 Munich, Germany; stephanie.combs@tum.de; 4 Department of Biosciences and Biomedical Engineering (BSBE), Indian Institute of Technology Indore, Khandwa Road, Simrol, Indore 453552, India; hemcjha@iiti.ac.in; 5 Key Laboratory of Drug-Targeting and Drug Delivery System of the Education Ministry, Sichuan Engineering Laboratory for Plant-Sourced Drug and Sichuan Research Center for Drug Precision Industrial Technology, West China School of Pharmacy, Sichuan University, Chengdu 610064, China; gaohuile@scu.edu.cn

**Keywords:** extracellular vesicles, biomolecular corona, post-secretory modifications, extracellular matrix

## Abstract

Extracellular vesicles (EVs) are an important mediator of intercellular communication and the regulation of processes occurring in cells and tissues. The processes of EVs secretion by cells into the extracellular space (ECS) leads to their interaction with its participants. The ECS is a dynamic structure that also takes direct part in many processes of intercellular communication and regulation. Changes in the ECS can also be associated with pathological processes, such as increased acidity during the development of solid tumors, changes in the composition and nature of the organization of the extracellular matrix (ECM) during fibroblast activation, an increase in the content of soluble molecules during necrosis, and other processes. The interaction of these two systems, the EVs and the ESC, leads to structural and functional alteration in both participants. In the current review, we will focus on these alterations in the EVs which we termed post-secretory modification and processes (PSMPs) of EVs. PSPMs can have a significant effect on the immediate cellular environment and on the spread of the pathological process in the body as a whole. Thus, it can be assumed that PSPMs are one of the important stages in the regulation of intercellular communication, which has significant differences in the norm and in pathology.

## 1. Introduction

Extracellular vesicles (EVs) are constantly present in the extracellular space (ECS) and mediate many processes, such as signaling induction and extracellular matrix (ECM) formation. In addition, they are of great interest as therapeutic agents and prognostic markers in medicine [1]. The differentiation of EVs into subtypes is a complex and non-trivial task. EVs can be classified based on their biogenesis, origin, biological function, or content [2,3]. However, the most widely used classification of EVs today is based primarily on their genesis. In general, there are three classes of EVs, known as exosomes, microvesicles, and apoptotic bodies. All three subtypes of EVs have a lipid bilayer membrane that surrounds their contents (i.e., proteins, mRNA, and miRNA). Exosomes and microvesicles are regularly released and taken up by cells to support intercellular communication. They have very similar properties and are very difficult to separate. One of the main differences between them is their mode of genesis. Exosomes are formed by the endolysosomal pathway following the invagination of endosomal membranes to form multivesicular bodies and are released after their fusion with the cell plasma membrane, while microvesicles are formed as a result of the cell membrane protruding outward [4]. Apoptotic bodies are typically the largest and, as their name suggests, are released by apoptotic cells. However, they are not simply remnants of apoptotic cells and play an important role in immune regulation and activate pathways that promote phagocytosis and the removal of dead cells’ bodies [5]. Due to the low specificity of the identified molecular markers, most studies consider total EVs. Moreover, the term “exosomes” is sometimes used to refer to all EVs. In addition, a fourth type of EV was recently identified—migrasomes, which are formed on the restriction fibers of migrating cells (Table 1) [6].

There are many unresolved issues in the life cycle of EVs. In 2019, Leonid Margolis and Yoel Sadovsky formed eight main directions and unsolved mysteries of the biology of EVs: (1) the size diversity of EVs, (2) the biogenesis of EVs, (3) the loading of EVs with molecular cargoes, (4) the specificity of released EVs to recipient cells, (5) the correlation of EVs and viral particles, (6) the delivery and unpacking of EVs in recipient cells, (7) the mechanisms of changing the biology of recipient cells under the influence of EV cargo, and (8) the functional significance of EVs in pathologies and normal state [13]. Most of the proposed tasks are already traditional for EVs researchers. However, we would like to add a ninth important direction to this list. The ninth direction is related to the transformation and modification occurring with EVs in the ECS. Newly formed EVs released by cells first enter the ECS, where they interact with its components, as well as the conditions existing there until they are absorbed by recipient cells. The ECS environment is quite dynamic and multicomponent. The main non-cellular participants in the ECS are considered to be the ECM, which is an interstitial fluid with various mediators of cellular communication [14]. Unfortunately, the processes occurring in the EVs remain largely a mystery. It is at this stage that various post-secretion processes and modifications (PSPMs) can occur with them. Recently, it has been shown that recipient cells can re-release internalized intact EVs following their initial uptake [15]. In this case, the internalization of EVs by recipient cells is not their final stage, since the re-released EVs retain their functional properties. We consider the stage of cargo release or extra/intracellular destruction of EVs to be the final stage. Thus, by PSPMs, we mean all events from their release to destruction that change the properties of EVs or control their interaction with recipient cells.

These transformations are another important stage of regulation of cellular interactions and allow for a more fine tuning of the existing extracellular microenvironment. The specific conditions of the cellular microenvironment contribute to the functional role of EVs. Extracellular systems determine various states that regulate cell biology. A striking example can be seen in the mechanisms of assembly of the ECM [16], where the interaction of different types of collagens with matrix formation occurs outside the cell through the participation of extracellular enzymes and other participants of the ECS in this process. Similarly, we assume that there may also be a large set of extracellular mechanisms that perform modifications with vesicles secreted by cells and thus form the post-secretome of the cell population both in vitro and in tissues and organs. In this regard, it seems extremely important to study the cell secretome as a tool for intercellular interactions in the presence of most components of the ECS and, accordingly, the low indicativeness of the vesiculome isolated from a monocomponent system as a characteristic of the cell status. In addition, because of the extensive PSPMs, it is necessary to more strictly standardize the methods for isolating EVs from samples.

Thus, this review aims to summarize the available data on the PSPMs and to form an applicable picture of the extracellular fate of EVs.

## 2. Alteration of the EV Surface

First of all, during secretion, EVs enter the extracellular space, which differs from the conditions inside the cell. Moreover, some EVs spread through tissue and are eventually found in a wide variety of biological fluids, including blood, lymph, saliva, ascitic fluid, synovial fluid, urine, etc. Such a sharp change in environmental conditions cannot but affect the properties of exosomes. A striking example is tumor stroma, which is characterized by hypoxia, high cellular density, interstitial pressure, and increased acidity. The influence of such factors in vivo, unfortunately, has been poorly studied. However, some aspects have been covered. The surface of EVs is an important functional part of these structures, which primarily determines their interaction with the cell environment and is in direct contact with extracellular conditions. The difference between these conditions and intracellular ones obviously leads to a change in the surface of EVs.

### 2.1. Alteration of Lipid Bilayers

The lipid composition of EV membranes and the plasma membrane of cells varies and often depends on EV biogenesis. The formation of MVBs as a precursor of exosomes is accompanied by the recruitment of cytosolic lipids of various origins such as organelle membranes, lipid droplets and lipoproteins [17]. This leads to the inclusion of triacylglycerols and cholesterol esters in the exosomal membranes [18]. In addition, increased levels of cardiolipin were found in exosomes, suggesting the contribution of mitophagy to the release of these EVs [19]. Enrichment with cholesterol and sphingolipids makes them similar to the detergent-resistant domains of lipid rafts [20]. The critical role of these lipid rafts in microvesicle biogenesis has been described in other reviews [21,22]. Quantitative changes in the lipid composition of EVs during release remain a mystery due to the complexity of the studies. Tore Skotland and colleagues, having analyzed a large amount of data, came to the conclusion that the quantitative lipid composition of EVs is heterogeneous and depends on many factors. Qualitative changes in the lipid composition of the EV membrane after secretion are also a likely but unproven event. We suggest that these phenomena may be mediated by lipid-modifying enzymes. Their involvement in EVs biogenesis has already been described, and it is likely that their activity may persist after release [23].

**Figure 1:**
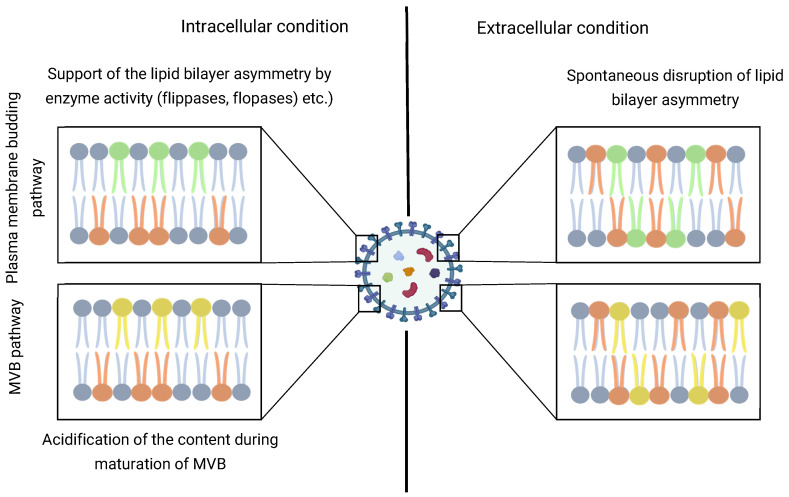
Changes in the asymmetry of the EV’s lipid bilayer during biogenesis. Green lipids—specific asymmetric for plasma membrane. Yellow lipids—specific asymmetric for MVB’s inner vesicles.

The asymmetry of the EV membrane is also an extremely interesting and mysterious issue. For EVs, membrane asymmetry is one of the stages of biogenesis, and it is especially important for microvesicles. The violation of lipid layer asymmetry is a less energy-consuming procedure compared to maintaining asymmetry, which is supported by the work of a large number of enzymes including flippases, floppases, scramblases, etc. [24]. These mechanisms are especially characteristic of the plasma membrane of cells. During the processes related to the budding and formation of microvesicles, the membrane asymmetry can also be preserved in EVs. In the exosomal pathway, the asymmetry at the of biogenesis can be maintained by the acidic environment and other factors within the MVB. Regardless of the biogenesis pathways, after release from the cell, the membrane’s asymmetry disturbances are possible due to the flip–flop transpositions of lipids in the bilayer, which can be accelerated by the action of physicochemical influences of the environment (Figure 1).

Asymmetry relative to phosphatidylserine (PS) shows a dual nature in EVs. For exosomes, PS is typically located in the inner layer [25], while for microvesicles and apoptotic bodies, it is concentrated in the outer one and is a signal for macrophagy [26]. However, some studies show that PS can also be located in the outer layer of the exosome membrane [27,28]. The authors associate this phenomenon with plasma membrane homeostasis under stress conditions and suggest the transition of PS to the outer layer during EVs’ biogenesis. Whether PS is able to move to the outer layer of the EVs membrane after release from cells or during EV storage remains a controversial issue. Similar controversial data concern the location of cholesterol in the EV membrane [29]. The overall asymmetry of the EV’s membrane is indeed poorly understood. However, extrapolating the data, one of the key factors in this process is the pH gradient. The pH gradient is probably one of the first events modifying EVs during and after secretion. This is especially true for exosomes, which are in an environment with a pH of 5.5 at the maturation stage in MVB and enter a more neutral environment of pH 7.4 during secretion. A reporter system for tracking exosome secretion was developed based on this pH change [30]. It was also previously determined that the increased acidity of the cellular microenvironment (as in the general cancer phenotype of about 6.5) causes an increased release of exosomes from the cell, but the mechanisms of increased secretion are unclear [31]. It is known that the absorption of EVs by recipient cells, with the exception of fusion, is mediated by their entry into endosomes, the maturation of which increases acidity, which induces their fusion with the endosome membrane and the release of EV contents into the cytoplasm. How acidic pH increases the tendency of membrane layers to fuse is not fully understood. Previously, protein interactions and pH conditions played a key role in the processes of MVB fusion in endosomes [32]. However, Morandi et al. showed that acidic pH is a prerequisite for the occurrence of this process [33]. Moreover, at the level of early MVB, the level of acidity inhibits the collapse of exosomes. Presumably, this is due to a violation of the asymmetry of the lipid bilayers. With different acidity inside the vesicles and the environment surrounding them, the flip–flop transposition of lipids within the bilayer is observed [33].

The direct effect of pH on membranes is partly explained by the acido-basic properties of lipids. The effects of acidic pH and temperature also affect the rigidity and fluidity of the lipid membrane and, accordingly, modulate the fusogenic properties of EVs [34]. The polarization of lipid vesicles is another process induced by a pH gradient in the region of low concentration of H + ions, after which the clustering and coalescence of ordered Lo domains occurs [35]. The application of knowledge about the effect of pH on the EV’s surface is extremely wide. The original tool was developed by Kim et. al. They developed a system of pH-dependent peptides to form regulated pores in lipid bilayer membranes [36].

In addition to asymmetry, the polarization, local deformation, and migration of EVs in a pH gradient were detected. Under these conditions, (i) the interaction of lipid heads to H+ or OH- groups as well as (ii) H+ and OH- gradients create conditions for chemical reactions of binding or catalysis [37]. EV migration was demonstrated in the work of Atsuji Kodama and colleagues using phospholipid GUVs as an example and modeling a pH gradient using NaOH microinjection with EVs starting to move toward the tip of the micropipette [38]. The mechanisms of vesicle migration activity under the action of a pH gradient are still a controversial issue, but this phenomenon is associated with the formation of a surface tension gradient on the surface of liposomes. The lipid membrane of EVs is a highly dynamic system that responds to environmental conditions. Many aspects contribute to the quantitative and qualitative composition of EVs as well as the asymmetry and polarization of the EV’s membrane even after secretion.

### 2.2. Formation of a Biomolecular Corona on the Surface of EV

**Figure 2:**
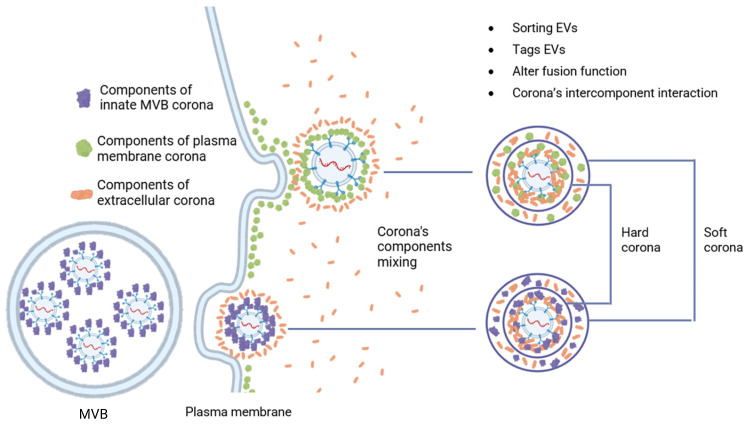
Formation of BMC on the surface of EVs in exosome and microvesicles pathways.

Another aspect of the impact of the extracellular environment can be considered—a high concentration of various soluble biomolecules. By analogy with other nanoscale structures (viral particles, nanoparticles, liposomes, etc.), it was suggested that biomolecular shells called protein corona can form on the surface of natural EVs [39]. The hypothesis was confirmed when it was shown that EVs, when incubated with blood plasma, are covered with a protein corona [40], which has functional significance and is involved in many intercellular communications [41]. The development of this hypothesis showed the participation of many soluble components in the formation of the corona around EVs under various conditions including proteins, lipids, polysaccharides, lipoproteins, glycoproteins, etc. That is why it is more correct to call this shell a biomolecular corona (BMC) [42,43]. Nucleic acids became an unexpected component of the BMC. Various studies prove the association of DNA with the EV membrane [44,45,46]. However, it remains unclear whether DNA is attached inside the cell during biogenesis or in the ECS after secretion. The formation of the BMC occurs at the stage of EV origin. In the exosomal pathway, the BMC is formed in the multivesicular bodies and surrounds all internal exosomes. When EVs are formed by separating the cytoplasmic membrane, the BMC initially associated with it is preserved [43]. In both cases, the structure of the BMC can be divided into two layers, where the first is characterized by a more rigid and low-mobility structure—«hard corona», while the second is more dynamic and changeable—«soft corona» (Figure 2) [39].

The consequence of the formation of a BMC around EVs is a change in the potential for fusion with recipient cells. It has been shown that coating with opsonins (LDL, IgG and C3b) facilitates uptake, while coating with dysopsonins (albumin, ApoA4, ApoC3, and clusterin) reduces uptake [47]. In addition, we assume no less participation of proteolytic enzymes of the ECM and chemical conditions of the pericellular space. In addition, the BMC has a number of important physiological actions such as healing processes and in immune modulation [48].

The potential for using the BMC in medicine may lie in its artificial modification or the creation of exosomes with a programmable BMC. These ideas were embodied in the work of Jun Yong Wu and colleagues in the creation of liposomes with increased permeability through the blood–brain barrier due to the coating of particles with the angiopep-2 protein (Ang), which protects the particles from destruction in the bloodstream [49]. Martin Wolf and colleagues selected a strategy for ligating antibodies to vesicles with the most optimal properties, and they formed a natural BMC [41].

#### 2.2.1. Post-Translational Modifications of Components

The BMC formed during the interaction of EVs with the environment creates favorable conditions for intermolecular interactions of its components, which can lead to posttranslational modifications (PTMs). The main PTMs of proteins found in EVs are phosphorylation, acetylation, oxidation, nitrosylation, methylation, glycosylation, ubiquitination, sumoylation, lipidation, isoprenylation, etc. Most of the modifications were studied in light of their participation in the packaging of EV cargo components [50]. Distinguishing intracellular PTM from the extracellular is a rather complex and non-trivial task.

Extracellular enzymes form the basis for the PTM of EVs. Many enzymes that carry out PTMs are found in the BMC. These enzymes can modify the components of the BMC, the EV membrane and other components of the ECS [51]. Thus, glycosidases (sialidase NEU3, NEU1) associated with the BMC are able to cleave chains of sialic acids; this process can be accompanied by the release of molecules, which was shown by the example of the release of neurotrophins during the destruction of retaining polySia [52,53]. Insulin-degrading enzyme (IDE) is another enzyme found in the BMC that is capable of performing many processes, including the degradation of insulin, amylin and glucagon as well as modulation of the ubiquitin–proteasome system, indicating it may regulate protein turnover and homeostasis [54]. In the BMC of EVs, the function of IDE has been shown in the degradation of extracellular Aβ by microglia [55]. MT1-MMP, a transmembrane protein found on the surface of many cell types, has also been detected in the membrane of EVs [56]. It is believed that it is integrated into EVs during biogenesis, especially in microvesicles budding from invapodia, where MT1-MMP concentration is the highest [57]. The modification activity in EVs is expressed in various actions. Thus, shedding by cleaving syndecan-1, ICAM-1 and CD44 leads to EVs release from the membrane surface. Cleavage of the host of soluble molecules including proMMP-2 and proMMP-13 indicates involvement in the regulation of activity of some soluble molecules [58].

Extracellular glycosylation is one of the probable PTMs occurring with EVs. As is known, fibronectin and tenestin are found on the surface of exosomes (the mechanism is described in the following reference [59]). These components can be glycosylated in the extracellular space similar to glycosylation in the ECM as part of aging processes and other pathologies [60]. Potentially, all natural processes of extracellular enzymatic and non-enzymatic PTM characteristic of natural ECM in addition to glycosylation can occur with the ECM components of EVs [61].

The biological significance of the described extracellular processes of PTM components of EV remains to be identified. On the one hand, they can be part of the natural extracellular maturation of EV. On the other hand, they can be part of pathological processes disrupting intercellular communication by analogy with disorders of healing processes mediated by glycosylation of the ECM and disruption of the interaction of integrin receptors with the ECM [62].

#### 2.2.2. Formation of Multimolecular Machine

The interaction of the components of the BMC is not limited to post-translational modifications. Some interactions lead to the formation of multimolecular functional apparatuses. Most studies related to the processes of interaction of soluble components of the BMC with EVs do not associate these processes with the BMC, but we believe that these processes occur in a single system of the EV’s BMC [63]. It is also worth noting that when studying the BMC, it is difficult to separate absorbed components from newly built-in components.

Nathalie Cloutier and colleagues showed that EVs can be a platform for the citrullination of autoantigens on the EV’s surface in the BMC by peptidylarginine deiminase 4 (PAD4) with the subsequent formation of mEV-IC (medium-sized extracellular vesicle-containing immune complex) with the deposition of immunoglobulins against citrullinated autoantigens inducing inflammatory processes [64]. A similar picture is reflected in the activation of immune cells by EV antigen-presenting cells. Secreted EVs in the ECS acquire MFGE8 (milk fat globule epidermal growth factor 8), interacting with phosphatidylserine. In this complex with EVs, MFGE8 can bind to integrins αVβ3 and αVβ5 of recipient cells and induce immune activation [65,66]. In addition to the previously described mechanisms of participation of the BMC of EVs in immune processes, participation in the activation of the complement system is likely. The C1q component is able to bind to the lipid negatively charged components of EVs via the highly cationic region C1qA14-26 or, as suggested, to the anionic proteins of EVs [67]. The further fate of such EV and C1q complexes is not yet identified; according to suggestions, activation of the classical pathway of complement activation may occur further. A similar event may also be mediated by the binding of immunoglobulins to antigens on the EV surface and the subsequent attachment of C1q [68]. Also, a more complex complement activation system based on the BMC was demonstrated on the basis of EV monocytes. Pentameric CRP (pCRP) binds to the EV’s membrane and undergoes conformational changes to form neoepitope-expressing (pentameric) CRP, which can subsequently bind to C1q and also activate the complement system [69].

The attachment of other immune components to EVs such as antibodies with the subsequent activation of cascades exhibits a dual nature. On the one hand, EVs that have receptors of infected cells on their surface neutralize the humoral response [70]. But on the other hand, such interaction protects against the antibody-dependent enhancement of infection. A similar picture was shown for *Zika virus* (ZIKV) and E-coated EVs [71]. In general, the picture shows that EVs are able to control the immune response to various pathological conditions.

Conformational changes in proteins are also an unlikely process for the components of the BMC. The participation of EVs in conformational changes during the interaction of proteins with their membrane was shown using the example of incorrect folding of αsyn when small EVs enhance synucleinopathy during the interaction of secreted αsyn with the EV membrane. The authors attribute the main contribution to this process to the lipid composition of EVs but also do not exclude the participation of membrane-associated proteins [72]. But we assume that these processes are associated with the BMC to no lesser extent.

Another example of the multimolecular apparatus on the surface of EVs are matrix vesicles. They will be discussed in more detail in the following sections. During their attachment to the ECM, the processes of hydroxyapatite synthesis in the vesicle lumen are launched and mediated by the work of various enzymes [73]. Views on the life cycle of MVs are still controversial, from the issue of their biogenesis to the destruction and accumulation of the mineral. The modern understanding of MVs is changing, and there is more and more evidence that MVs can be formed in different ways [73]. According to one of them, secreted exosomes can become MVs, and at the stage of origin in endosomes, they acquire stimuli for mineralization [74,75]. Comparison of the proteomes of MVs and apical microvilli, as the main site of their biogenesis, revealed the absence at the stage of origin of some participants, including chondroitin sulfate proteoglycan 2, cysteine-rich angiogenic inducer 61, immunoglobulin J chain, macroglobulin α2, syndecan 2, syntaxin 4A and vitronectin [76]. This allows us to assume that these components are acquired by MT already in the VKP and are part of the BMC.

## 3. Intervesicular Extracellular Interactions

During the interaction of EVs with each other, a variety of events can occur. Direct studies of these processes, unfortunately, are insufficient to build a complete picture. Extrapolating from liposome studies, it can be assumed that this interaction can lead to three possible outcomes: repulsion, adhesion with the preservation of isolated cavities, and the unification (fusion) of EVs. The repulsion of membranes occurs in aqueous solutions due to the interaction of polar groups of phospholipids and hydration shells [77]. This process provides protection against the collapse of cellular organelles.

### 3.1. Mutual EVs Sticking

The adhesion of EVs is a documented outcome of their interaction, which is associated with the interaction of the EV’s BMC with each other, which can lead to coagulation and the formation of thromboses [78].

In addition to the participation of the BMC, the adhesion of EVs can occur at the level of interaction of lipid membranes. A special case of such adhesion of EVs can be considered the activity of platelets. Platelets, as a special group of apoptotic bodies, as well as microvesicles, are characterized by a negatively charged lipid membrane due to the high content of PS. Due to this, when interacting with coagulation factors (factors VII, IX, X and prothrombin) containing positively charged γ-carboxyglutamic acid and anticoagulant proteins S and C, their adhesion occurs with the formation of a thrombus [79].

It should be noted that the adhesion of EVs can be controlled by the composition of the membrane. The anticoagulant properties of EVs can be associated with the presence of the C protein receptor on the membrane, and when it interacts with the ligand, the anticoagulant properties are preserved [80]. Moreover, due to the content of proteins on the membrane, TFPI (Tissue Factor Pathway Inhibitor) PAI-1 (Plasminogen Activator Inhibitor 1), EVs can exhibit fibrinolytic activity [81,82].

### 3.2. EVs Extracellular Fusion

The fusion of exosomes with each other is a possible process that we hypothesize. Canonical mechanisms of EV fusion are associated with absorption by recipient cells and their subsequent events in the cell. The main pathways of cellular absorption are endocytosis, pinocytosis, phagocytosis, and membrane fusion [83]. However, these mechanisms are strictly regulated by various systems and practically do not occur spontaneously. The mechanism of EV fusion is primarily accompanied by membrane fusion. The spontaneous convergence of membranes with the subsequent fusion of lipid layers in the body, as mentioned earlier, is limited by repulsive forces. To overcome this barrier and induce membrane fusion, it is necessary to ensure a convergence of membranes of less than 20 Å [77]. Therefore, such processes must be mediated by various auxiliary mechanisms [84].

We assume that in vivo exosome fusion with each other with cargo aggregation can occur. An obvious consequence of such an interaction is an increase in the heterogeneity of the population of the EVs by size. Unfortunately, there is no direct evidence of these fusions of EVs in vivo or in vitro. Next, we will try to extrapolate data from works with artificial liposomes to natural EVs.

In fact, there are two approaches to overcome the limitations of lipid bilayer fusion. The first is associated with a decrease in the membrane charge or a decrease in the contact of lipid layers with water using chemical fusogens. The second is based on the use of protein/peptide fusogenic agents [85]. Both of these approaches are used in the MFHE technology.

The first approach can be realized by various mechanisms in vivo. As described earlier, an acidic environment and high temperature can enhance the fusogenic properties of EVs and induce their fusion by mediating an increased layer flip–flop transposition of lipids and decreased membrane charge [33]. Unfortunately, there is no direct evidence of this process. However, there are interesting data on similar processes in vitro when creating artificial vesicles. An interesting mechanism of exosomal fusion was recently demonstrated in the work of Sumit Kumar, where the controlled fusion of exosomes was provided by the interaction of catechol-integrated membranes with each other mediated by the inclusion of metal ions [86]. It is worth mentioning that in the case of the first approach, the BMC of EVs will interfere with their spontaneous fusion. However, with a decrease in its density and other factors, such an event is possible.

The second approach to membrane alteration based on fusogenic agents can also be observed in vivo. A striking example of such an auxiliary mechanism for EV fusion can be fusogens. Fusogens are a group of proteins that provide controlled membrane fusion by bringing lipid layers closer together (to a distance of ~1 nm) [87]. For activity, EV’s fusogens require the availability of a specific receptor on the acceptor membrane, for example syncytin-2 and syncytin 2-specific receptor (HFSD2a) [88]. These processes have not been shown for intervesicular to interaction, but they remain likely.

In practice, EV fusion using fusogens is used to create the previously described hybrid therapeutic vesicles. Thus, due to the expression of baculo-viral envelope protein gp64 on the EV membrane, it was possible to achieve pH-controlled EV with artificial liposomes [89]. A similar mechanism was used in the work of Ilya Zubarev, where a genetically engineered insertion of the viral spike receptor increased the internalization of exosomes with cells [90].

The properties of membranes for fusion are used in the creation of artificial liposomes of various sizes. The most common technique for preparing a suspension of liposomes based on their fusion is spontaneous fusion under controlled conditions, and the characteristics of the resulting liposomes depend on this process. The direct use of the fusion of EVs is used in the creation of hybrid therapeutic exosomes called membrane fusion-based hybrid exosomes (MFHEs) [91]. The main components for MFHEs are liposomes loaded with therapeutic agents and EVs. Due to this composition, the procedure for packaging therapeutic agents into the cavity of exosomes is simplified while maintaining high biocompatibility. The main methods of MFHE synthesis are freeze–thaw cycles, incubation, PEG incubation, and extrusion [92].

The question of the consequences of such a fusion of EVs also remains open. It is obvious that during fusion, the size of EVs increases equivalent to the previous EVs. The mixing of cargoes can induce their interaction with a variety of outcomes.

**Figure 3:**
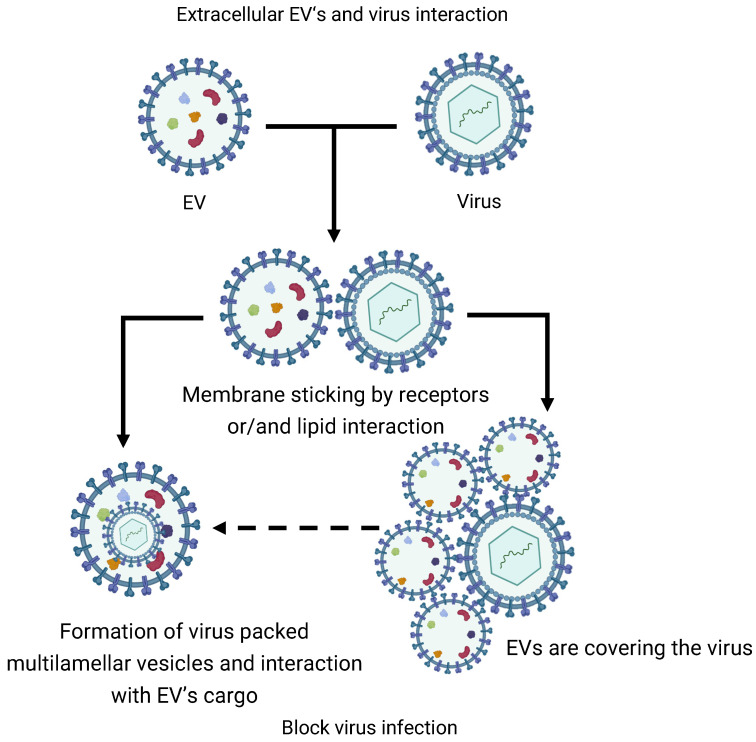
Scheme of the proposed mechanism of inhibition of viral infection by EVs.

In addition to mutual interaction, EVs can interact with other circulating structures of the ECS such as viral particles. To a certain extent, virions are structurally and even functionally similar to EVs [93]. Thus, it was determined that EVs from symbiotic vaginal lactobacilli inhibit human immunodeficiency virus HIV-1, and inhibition occurs at the level of the virus, since EVs block gp120 receptors on the surface of the virus, stopping their attachment to cells [94]. Similar data were obtained for ACE2-positive EVs and SARS-CoV2 virions [95]. Unfortunately, the mechanisms of this blocking of the interaction of virions with target cells have not been determined. Probably, EVs stick to virions and, upon such close contact, they collapse and the virion is packaged into the EV cargo (Figure 3). This results in the formation of multilamellar or multivesicular EVs, which are attracting increasing attention. The formation of such complex structures in vivo is associated with biogenesis mechanisms and is described in more detail in other reviews [96,97]. However, we assume that these structures can also be formed extracellularly during intervesicular interactions in the ECS.

Studies of these processes can shed light not only on the functioning of cells and their communications but also help to develop new approaches to therapeutic intervention in various pathological conditions. In addition, they will help researchers optimize work with EVs in the selection of isolation methods and the processes occurring with them during storage.

## 4. Volume Alteration and Deformation of the EV

Osmotic processes are an inevitable and integral part of the EV life cycle. The critical role of osmotic characteristics in the processes of exocytotic fusion of vesicles with the cell membrane was shown using the full collapse and kiss-and-run models [98]. In addition to their functional significance, osmotic processes maintain the EV structure. At the biogenesis stage, extracellular osmotic hypertonic stress can quickly cause vesicle compression while maintaining a constant neurotransmitter concentration, which suggests an osmometric function of vesicles [99].

**Figure 4:**
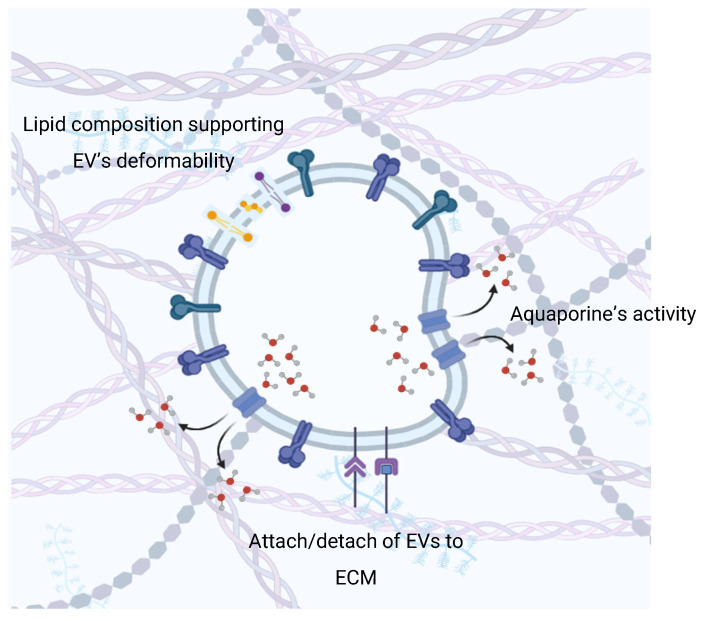
Volumetric changes and deformability of EVs in the ECM.

These processes play a critical role in the distribution of EVs in tissues, since the porosity of the ECM network does not allow large EVs exceeding the pore size to pass [100]. On the one hand, this problem is solved by the ECM remodeling agents of EVs. On the other hand, dynamic changes in EVs are no less important for ensuring distribution. Deformation and change in EVs is a necessary process in their distribution in the extracellular space (Figure 4) [101].

A key role in volumetric changes in EVs is given to aquaporins (AQPs). AQPs are a family of small (~30 kDa) membrane-spanning proteins that provide rapid, passive, and bidirectional movement of water, small neutral solutions, and some ions into and out of cells in response to osmotic or concentration gradients. To date, AQPs are considered as one of the key transmembrane osmosensors of the cell along with mechanosensors of ion channels [102]. Orthodox AQPs 1, 2, 3, 4, 5, 7 and 9 were found on the surface of mammalian EVs [103]. Their role in EVs is associated with adaptability to various osmolar conditions of the extracellular environment to maintain the integrity of vesicles. The assumption is indirectly proven by functions in cells. For example, AQP1 moves into exosomes as RBCs mature [104]. In addition, the critical role of AQP6 in EVs functionality was shown in the synaptic vesicle model. The swelling of synaptic vesicles potentiates their fusion at the cell plasma membrane and is required for the expulsion of intravesicular contents [105,106]. Thus, the disruption of EV swelling processes mediated by vesicle acidification by H+-ATPase and AQP6 function led to the disruption of neurotransmission on account of the AQP-6-mediated gating of water into synaptic vesicles [107,108]. Another role of AQP in EVs was shown in protecting the latter from lysis. The inhibition of AQP5 functions with antibodies or HgCl_2_ (AQP5 inhibitor) caused the swelling and subsequent lysis of parotid secretory granules in isoosmotic KCL solution [109]. In models of secretory vesicles, the role of AQP was shown in the processes of exocytosis (fusion with the plasma membrane and release of vesicle contents) and vesicle swelling as one of the key stages of regulation of this process [110]. We suggest that a similar role of AQP can be observed in EVs, especially in the processes of their absorption by recipient cells by fusion or fusion with the membrane endosomes following absorption. Thus, we conclude that the osmosensory role of AQP in EVs provides (i) EV distribution in the ECS, (ii) EV functional properties, (iii) the maintenance of EV integrity, and (iv) the regulation of absorption by recipient cells. Protein factors can also play an important role in EV deformation. Thus, α-syn was shown to participate in the processes of EV release/absorption, EV deformation and EV fragmentation [111]. Artificial DOPC:DOPS liposomes begin to fragment upon interaction with α-syn only when monosialotetrahexosylganglioside (GM1) is included in the liposomes. The putative mechanism of this phenomenon is associated with the interaction of α-syn with GM1 liposomes, which, due to the large hydrophilic heads and short tails of the latter, induces a deeper immersion of α-syn into the membrane [112]. This demonstrates the dual role of α-syn in neuronal signal transmission. On the one hand, it facilitates the endo/exocytosis of EVs; on the other hand, it disrupts their traffic [113].

Smaller deformation changes in EVs can occur under the influence of a pH gradient. As described earlier, liposome migration is observed during such a gradient. This process is carried out with the deformation of vesicles, and the stretching of spherical liposomes to a drop-shaped form occurs toward the highest concentration of OH- anions [38].

In addition to passive regulation, it is assumed that the dynamics of EVs can be controlled by the active activity of cytoskeletal components found in large EVs (LEVs). This EV subtype is characterized by large sizes up to several micrometers, negative staining for DAPI, the presence of intact organelles and a functioning cytoskeleton [114]. The presence of an active cytoskeleton allows these LEVs to deform and exhibit mobility, and the presence of adhesion molecules ensures their attachment to matrix components. The authors associate the role of these LEVs with the transfer of subcellular organelles such as mitochondria and increased tumor resistance. In our opinion, LEVs can also function as a utilizer of large cellular components, such as organelles. Such large sizes make them less accessible to cells [115]. Their mobility and deformability ensure movement into the bloodstream, which suggests further utilization [116]. In smaller EVs, cytoskeletal components were also found that caused a change in shape other than spherical. Similar subpopulations of EVs were found in human ejaculate and in the human mast cells of the HMC-1 line [117,118]. This phenomenon is associated with the protrusive pathway of EV biogenesis and, as a consequence, the presence of actin filaments in them [119]. In this case, whether the altered shape of EV is simply a consequence of the presence of actin filaments or some advantage over the spherical one is unknown and remains a controversial issue.

## 5. Attachment of the EV to Extracellular Matrix

The multicomponent nature of the extracellular space suggests that secretory exosomes interact not only with recipient cells. One of the best-studied examples is the interaction of the extracellular matrix with exosomes to deliver extracellular matrix proteins.

It is already becoming indisputable that EVs are an important integral and functional component of the ECM [120]. The properties of the ECM have a broad impact on EVs at all stages of their life cycle. For example, at the biogenesis stage, the rigidity of the matrix and the number of cell contacts with it inversely determine the intensity of MVB fusion with the plasma membrane and, accordingly, the release of exosomes [121].

In this section, we focused on the attachment of EVs to various participants of the extracellular space, including the ECM, which is associated with further absorption by recipient cells. Many aspects of the interaction of EVs with extracellular matrices have already been described in an outstanding review of Koushik Debnath et al. [101]. The main components of the extracellular matrix are collagens, proteoglycans (PGs) and glycosaminoglycans (GAGs), elastin and elastic fibers, laminins, fibronectin and other proteins, such as matricellular proteins [122]. The attachment of EVs to the ECM can be roughly explained by two purposes: the first is associated with the delivery of ECM remodeling agents, while the second is associated with the subsequent internalization with cells or both of these purposes simultaneously [123]. Thus, it was shown that exosomes of dermal fibroblasts in three-dimensional cultivation contain, in addition to a large set of extracellular matrix components, many stimuli for the migration, proliferation, and inflammatory reactions [124]. From this point of view, the ECM with adsorbed EVs can be considered as an attractant (Table 2).

Previously, we described the mechanisms of EV passage through smaller pores of the ECM, but cases of EV attachment to the ECM have been proven [94]. The direct interaction of EVs with the matrix can be mediated both by the interaction of receptor proteins with the matrix and directly by the lipid membrane; the subtleties of the biophysical interaction of EVs with the ECM are described in detail in the review [101].

A special subgroup of EVs closely related to the ECM, which was mentioned earlier, are the matrix vesicles (MVs) first described in connective tissues [125]. Later, the mechanism of MV formation was described as ‘Verdämmerung der Zellen’ [126], which describes their emergence from the plasma membrane of mineral-forming cells such as chondrocytes, osteoblasts and odontoblasts [127]. MV produced by chondrocytes and bone cells are released from the ECM only when treated with collagenase, which is explained by their high affinity for collagen fibrils and high activity; in addition, they have a special mechanism of biogenesis associated with outward budding from the apical microvilli of mineralization-competent cells and high activity of tissue non-specific alkaline phosphatase (TNAP) as a key enzyme of biomineralization [73]. The mechanism of EV attachment to the ECM remains completely undefined. The most promising components retaining MVs are considered to be collagen type I [128]. The anchoring components of MVs holding them on collagen fibers are considered to be AnxA2, AnxA5, AnxA6 and TNAP [73]. In support of this, a recent study of the mechanisms of MT attachment using vesicle-biomimetic proteoliposomes containing TNAP and annexin A5 (medium-sized EV protein) showed binding to collagen fibrils via annexin A5 only in the absence of TNAP. The authors associate the results with the dynamics of MT binding processes to the ECM, which is caused by the interaction between MT components, both native and acquired [129].

The population of MVs associated with the ECM obtained during tissue decellularization was termed matrix-bound nanovesicles (MBVs) [130]. Notably, in this case, MVBs have reduced or no canonical exosome markers (CD63, CD81, CD9, and Hsp70) [131]. The mechanism of their attachment to the ECM was not described. The study of MBVs cargo and free MVs showed differences in protein components and miRNAs. MBV-associated miRNAs are associated with organ development, while free MVs cargo was associated with cell growth, development, and proliferation. In addition, differences are observed in the lipid composition. MBVs are characterized by a high content of cardiolipin, which suggests the involvement of the mitochondrial apparatus in their biogenesis [130].

The biological significance of EV adhesion to the ECM requires additional study. However, there is evidence that such an interaction can mediate absorption by recipient cells. Thus, it was shown that extracellular fragments of fibronectin interact with heparan sulfate receptors on the EV surface and mediate the interaction of the EV and fibronectin complex with myeloma/bone marrow stromal cells, thereby controlling EV absorption [132]. The control of uptake has also been shown by the interaction of EVs with soluble heparin, which is thought to block EV receptors and inhibit their binding to recipient cells as well as induce their aggregation [133]. The attachment of EVs to ECM components may mediate the degradation and remodeling of the latter, leading to altered cell invasion and migration [134]. Unfortunately, the mechanisms of remodeling of such attached EVs on the ECM have not been identified. Presumably, this may be associated with the activity of GMC components and transmembrane components such as MT1-MMP, IDE, and others described earlier. Another ECM remodeling agent of EVs can be considered aggrecan kinases providing remodeling of the aggrecan-rich ECM, which are characteristic of cartilage tissues and brain tissues [135].

The processes of EV attachment to the ECM can be inhibited by the formation of the BMC described earlier. Moreover, matrix-remodeling enzymes such as MT1-MMP, metalloproteinases (MMP2, 3, 9, 13, 14), adamazines (ADAM-10, ADAM-17, ADAMTS-5, ADAMTS-8), hyaluronidase, and heparonase were found among the components of the BMC, which are capable of releasing ECM-attached EVs and other complexes and molecules [134].

The use of the property of exosomes to attach to skeletal proteins was found in regenerative medicine when creating cell-free exosome-laden scaffolds [136]. We suggest that the uptake of exosomes by non-cellular components of the extracellular matrix may also be a tool for exosome selection and the evasion of recipient cells from interactions with them. In addition, the extracellular matrix network may control the spread of EVs through high density. As described earlier, the difference between the pore diameter of the ECM and EVs does not allow the free spread of the latter [100].

The attachment of EVs to ECS components is not limited to interaction with the ECM. However, other EV acceptors are less studied. Another likely acceptor of circulating EVs can be considered NETosis traps formed during neutrophil-specific programmed death. As mentioned earlier, the EV membrane is able to associate DNA on itself [44,45,46]. This suggests that circulating EVs can attach to NETosis networks. The nature of EV binding to DNA was not described. One of the probable models is the interaction of nucleic acid binding protein on the EV surface with circulating nucleic acids [137]. In addition to membrane attachment, this event can be provided by dsDNA receptors TLR.

## 6. Extracellular Disrupt of the EV

The involvement of EVs in extracellular processes is an indisputable fact. This section will discuss the main processes and mechanisms associated with extracellular EV destruction. EV destruction with the subsequent release of cargo might have different consequences.

As mentioned earlier, EVs are a supplier and agent for the construction and modeling of the ECM. Collagen components of the ECM, according to the canonical mechanism, are too large to be packaged into vesicles [138] and are therefore secreted into the extracellular space in the form of tubular structures emanating from the Golgi apparatus to the plasma membrane [139]. Meanwhile, non-collagenous water-soluble ECM components (fibronectin, tenascin, etc.) are poorly studied and can potentially be secreted by EVs. Knockouts of genes associated with COP2 vesicles do not affect the secretion of fibronectin components, which suggests other secretion pathways [140,141]. With regard to Tenascin-C, it was shown that exosomal secretion is the main source of its delivery to the extracellular space [59]. During the endosomal absorption of fibronectin and tenascin molecules during cell migration, their relocation to the surface of exosomes with subsequent release from the cell is observed [132,142,143].

Biomineralization and calcification is another process mediated by the extracellular destruction of EVs. As previously described, matrix vesicles responsible for these processes have a number of features. Their life cycle is associated with the accumulation of phosphates and calcium ions inside the lumen and the subsequent synthesis of hydroxyapatite [73]. This is synthesized as a “needle-shaped” or “ribbon-like” structure and, upon reaching a critical size, it ruptures the MV membrane and interacts with collagen fibrils [144]. Also, the probable mechanism of MV rupture is associated with the activity of phospholipases, which is described in detail in the review by Saida Mebarek et al. [145].

Another isolated example of the extracellular destruction of EVs can be considered the rupture of migrasomes. The first study of migrasomes showed both a hollow and multivesicular structure of migrasomes called a pomegranate-like structure [146]. The life cycle of migrasomes implies a probable rupture or loss of tightness in the mature state with a sequential release of vesicles, chemokines, cytokines, and growth factors and other cargo components [147]. The mechanism of this stage of migrasome rupture, unfortunately, remains unclear. Yaxing Zhang and colleagues identified the study of the mechanisms of migrasome rupture as one of the key areas of research on these organelles. This in turn determines the functional role of migrasomes in intercellular communication [148]. We assume that this process may be associated, on the one hand, with the activity of extracellular phospholipases, similar to the MV rupture [145]. On the other hand, cell-regulated mechanisms recruited to the migrasome to induce rupture may be responsible for this mechanism. Spontaneous rupture, in our opinion, is unlikely, because the migrasome structure itself is quite resistant to degradation and the loss of tightness. This was shown during the development of vaccines based on engineered migrasome-like vesicles (eMigrasomes). The resulting eMigrasomes were morphologically identical to native migrasomes and showed an impermeability to agents that mimic the size of the normal protein, and they retained their number and morphology even after storage for 14 days at room temperature. The authors attribute high stability to the membrane enriched with cholesterol and high rigidity, because when cells were treated with selective extracts of cholesterol from the plasma membrane—MβCD, the resulting eMigrasomes ruptured within 30 min [149].

In the applied aspect, researchers often face the goal of avoiding extracellular destruction and preserving EVs for research or therapeutic purposes. The significance of the non-programmable extracellular destruction of EVs may be associated with the disruption of intercellular communication. However, given that EVs, in addition to mediators, are also a tool for recycling cellular components, this event may be associated with the clearance of waste fragments. It is indeed a difficult task to distinguish secreted soluble factors from those released from destroyed EVs. But research in this area can shed light on many issues.

## 7. Therapeutic Application of EVs

In the past decade, researchers have been exploring the potential of EVs as diagnostic biomarkers and as therapeutic tools. EVs are natural drug delivery systems for the treatment of different diseases including cardiovascular diseases, neurological diseases, skin disorders, lung diseases, osteoarthritis, damaged tissue repair, and other diseases [150]. The diverse functions and characteristics of EVs highlight their importance in a wide range of biological processes and the potential for their use in a variety of clinical applications. Stem cell research and the global COVID-19 pandemic have given impulse to the development of cell and cell-free therapy for infectious diseases. The application of EVs is a promising treatment strategy that allows solving the problems associated with the safety of cell therapy and increasing its effectiveness [151]. It has been established that EVs have the same immunomodulatory and anti-inflammatory and other effects as their parental cells. However, there are different mechanisms underlying the interaction of various EVs derived from different types of mesenchymal stem cells (MSCs) with immune cells. For example, EVs derived from bone marrow, adipose tissue and umbilical cord have an influence on the T cells, increasing IL-10 and TGF-beta [152], through regulating TGF-beta and PGE2 [153] and through the COX2/PGE2/NF-kB signaling pathway [154].

The regenerative potential of EVs is mainly explained by the regulation of cell proliferation, differentiation, angiogenesis apoptosis, and inflammation [155]. The precise mechanisms underlying the therapeutic effect of EVs remain to be fully elucidated. However, several factors have emerged as promising candidates for mediating regenerative potential: microRNAs (miRNAs), messenger RNAs (mRNAs), and different proteins.

The intravenous administration of EVs is the one of the most used methods for laboratory research and clinical trials. Despite the cell-free therapy effectiveness, there are several disadvantages of using EVs for the treatment of various diseases, such as their rapid removal from the bloodstream and low amounts of effective substances. In addition, the spatiotemporal dynamics of EVs in vivo have remained largely unresolved due to the lack of a suitable method. Conventional EV-labeling methods are mainly based on fluorescent (FL) dyes (PKH2, PKH26). However, these lipophilic FL dyes can spontaneously form nanometer-sized micelles, resulting in the incorrect detection of EVs [156]. In addition, it has been shown that the increase in the size of PKH-labeled EVs compared to their unlabeled counterparts may affect their biodistribution, transport, and uptake by recipient cells [115].

A study by Rodriguez et al. demonstrated that the use of ex vivo stimulation of whole blood with fluorescently and genetically labeled EVs is a reliable, sensitive, and physiologically relevant model suitable for studying blood cell–EV interactions. It has been shown that EVs with pLenti-palmGRET reporter plasmid encoding the dual reporter palmitoylated EGFP-nanoluciferase protein were detected in association with CD20+ B cells within 1 min of intravenous administration [157]. Moreover, vesicles from two different cell sources have been shown to interact with CD20+ B cells to an apparent advantage. It remains to be seen whether this tropism is supported by EVs from other cell types.

New evidence shows EVs administrated into the bloodstream become coated with a “corona” [39,43] of proteins and other molecules that can influence the particles–cellular interactions. It is possible that non-surface-engineered EVs must adsorb plasma components to mediate interactions with B cells. B cells constitute a vital component of the adaptive humoral immune system. However, it is unclear whether the surface interaction or internalization of EVs by B cells occurs. Is there a change in the functional state of B cells?

## 8. PSPMs’ Contribution to the Optimization of Protocols for Working with EV

The main tool for standardizing work with EVs is represented by ISEV guidelines and recommendations [158,159]. However, even within the framework of these recommendations, the results of EV isolation can vary greatly and create contradictory research findings. The problem of separating EVs into subpopulations of exosomes, microvesicles, etc. has not yet been solved in practice. In this regard, it is worthwhile to be careful when naming the fractions of isolated EVs.

We conditionally divide EV studies into structural and functional. The former are focused on the study of the load, composition and structure of EVs; for this area, it is most important to obtain well-purified EVs in large quantities. The second area is associated with the study of the role of EVs in the functioning of the body (intercellular communication, secretion, modulation of the ECS, etc.), for which it is important to preserve the native properties of EVs. A no less significant contribution is made by the methods of storing EV preparations. Accordingly, we believe that the method of EV isolation and storage should be selected based on the objectives of the study. In confirmation of this, it was shown that the BMC of EVs can regulate the uptake by recipient cells and even retarget them to a specific group of cells [160,161]. The effect of isolation methods on the preservation of EV BMC has been well studied to date. Unfortunately, at the moment, a method for isolating EVs with the best preservation of BMC has not been determined. However, it is known that the most common methods such as ultracentrifugation and size-exclusion chromatography showed an extremely low preservation of BMC [41]. It is noteworthy that the BMC of EVs after isolation by ultracentrifugation can be restored with the preservation of physiological activity by incubating the isolated EVs with a late fraction of ultracentrifugation containing soluble components [162]. Presumably, this stage can become a solution to the serious problem of maintaining BMC.

Structural studies of EVs are not so demanding on the methods of EV isolation. The main problem is the contamination of samples with various cellular components or soluble molecules. A comparison of four methods of EV isolation according to the criteria of biophysical characteristics called ‘EV-nano-metrics’ from a condensed medium of breast cancer cells—(i) ultracentrifugation, (ii) density ultracentrifugation, (iii) size exclusion or immunoaffinity, and (iiii) precipitation—showed controversial results. In terms of the amount of isolated EVs, precipitation isolation turned out to be the most effective; however, atomic force microscopy showed the highest number of aggregates and polymeric residues in this case [163]. One of the approaches to overcome the limitations of isolation methods is a combination of methods, so the use of ultracentrifugation followed by size-exclusion chromatography (SEC) showed higher proteome preservation compared to the use of each method separately [164].

We believe that the development of new methods for isolating and storing EVs will help researchers optimize these procedures. One of the promising solutions for the isolation of EVs can be considered microfluidic isolation from various environments. This tool allows for both labeled receptors-dependent isolation [165] and size-dependent isolation [166]. It is equally important to expand the methods for studying EVs. One such promising method is spectroscopy, which provides a comprehensive molecular fingerprint of EVs without requiring sample labeling or complex pre-processing steps that can induce artifacts [167].

The solution to many technical problems can be the study of EVs in situ. But, unfortunately, the methods for implementing these studies are very limited and difficult to create. In vivo and in vitro studies of EVs without isolation have received a strong impetus in recent years, which is associated with the development of methods for intravital visualization. More and more relevant reporter systems are appearing that allow tracking single EVs [168].

## 9. Conclusions

Post-secretory modifications of exosomes are an obvious but unfortunately poorly understood mechanism of intercellular regulation. We assume that PSMPs, due to their dependence on the entire cell population, are a promising marker of pathological processes. Their biological role is quite difficult to predict. Most likely, they are part of the normal process of the post-secretory maturation of EVs and an additional stage in the regulation of intercellular communication. From this point of view, a balance arises between the release, absorption and removal/destruction of synthesized EVs.

In addition to regulating the absorption of EVs, PSMPs presumably provide an extracellular maturation of EVs, which is critical in the physiological effect on recipient cells.

Applied research in this area has somewhat overtaken fundamental research. Thanks to the exosome-based target delivery system, it has become feasible to create more stable and resilient liposomes. A deeper understanding of the post-secretome mechanisms can become a source of new methods of cell influence and regulation for the treatment of various diseases. In addition, research in this area will help to better understand the processes occurring with therapeutic nanocarriers, in particular artificial EVs, in the human body and optimize them. Some examples have been described earlier in the relevant sections.

The cumulative study of cell ‘vesiculoma’ is often interpreted as an indicator of the cell status. However, when accepting the fact of post-secretory processes, it becomes obvious that the secretome reflects the state of the entire cell population and the conditions of the ICP. When studying extracellular vesicles of liquid biopsies such as blood, urine, bronchial lavages, etc., the number of participants potentially changing their characteristics is so large that it becomes difficult to isolate their initial characteristics.

Based on the available data, we have identified only a few aspects of PSPMs such as changes in the EV surface, interexosomal interactions, volumetric changes and deformation, the attachment of EVs to ECS participants, and extracellular destruction. Needless to say, the real picture is much more complex and still emerging.

## Figures and Tables

**Table 1:** Characteristics of EV subtypes.

Subtype	Size	Surface Marker	Biogenesis	Ref.
Exosomes	50–150 nm	Classical panel: CD9, CD63, TSG101, Alix, Hsp70 Potential specific markers: LAMP1, LAMP2, Rab27, Rab27b	Inward budding of a late endosome, also known as a multivesicular body (MVB) with subsequent fusion with the cell surface and realese of exosomes into the extracellular space	[7,8,9]
Microvesicles/Ectosomes	100–500 nm	MT1-MMP, GP1b, GPIIb/GPIIa, P-selectin, Integrin, Mac-1	Direct budding out of a cell’s plasma membrane and shedding into the extracellular space	[10]
Apoptotic bodies	1000–5000 nm	Annexin V, Thrombospondin, C3b	Remnants of apoptotic cells	[11]
Migrasomes	500–3000 nm	TSPAN4, NDST1, PIGK, CPQ, EOG, IntegrinA5	Formation of primary swelling on the retraction follicles of migrating cells and filling with migrasomes with the formation of a pomegranate like structure (PLS)	[6,12]

**Table 2:** Examples of crosslink EVs to ECM.

	EV’s Membrane Protein	EV’s Lipid Membrane
Covalent binding to matrix	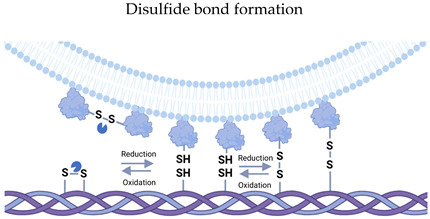	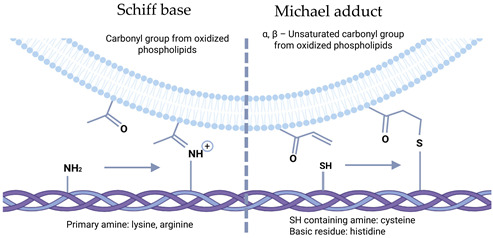
Non-covalent interaction of EVs with the matrix	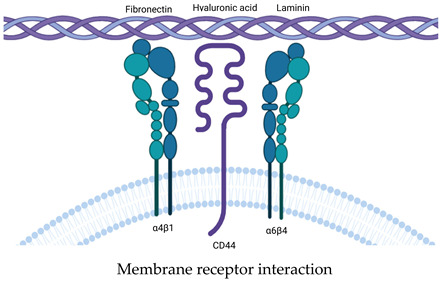	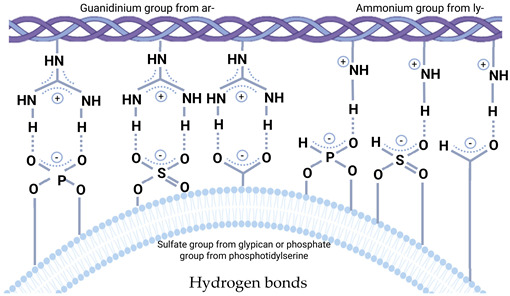

## Data Availability

The datasets used and/or analyzed during the current study are available from the corresponding author Maxim Shevtsov on reasonable request.

## Abbreviations

ACE2	angiotensin-converting enzyme 2
AQP	aquaporin
BMC	biomolecular corona
COP2	Coat Protein Complex II
COX2	cyclooxygenase-2
DOPC	1,2-dioleoyl-sn-glycero-3-phosphocholine
DOPS	1,2-dioleoyl-sn-glycero-3-phospho-L-serine
ECM	extracellular matrix
ECS	extracellular space
EV	extracellular vesicles
GUV	giant unilamellar vesicles
HIV	human immunodeficiency virus
LUV	large unilamellar vesicles
MBV	matrix bound vesicles
mEV-IC	medium-sized extracellular vesicle-containing immune complex
MFHE	membrane fusion-based hybrid exosomes
MMP	matrix mettaloproteinase
MSC	mesenchymal stem cell
MV	matrix vesicles
MVB	multivesicular bodies
MβCD	methyl-β-cyclodextrin
NF-kB	nuclear factor ‘kappa-light-chain-enhancer’ of activated B-cells
PAI-1	Plasminogen Activator Inhibitor 1
PGE2	prostaglandin E2
PS	phosphatidylserine
PSMP	post-secretion processes and modifications
SEC	size-exclusion chromatography
SUV	small unilamellar vesicles
TFPI	Tissue Factor Pathway Inhibitor
TGF-beta	transforming growth factor beta
TNAP	tissue-non-specific alkaline phosphatase
ZIKV	*Zika virus*
